# Topological analysis of tetracyanobenzene metal–organic framework

**DOI:** 10.1038/s41598-024-52194-1

**Published:** 2024-01-20

**Authors:** Ibrahim Al-Dayel, Muhammad Faisal Nadeem, Meraj Ali Khan

**Affiliations:** 1 Department of Mathematics and Statistics, College of Science Imam Mohammad Ibn Saud Islamic University (IMSIU), P.O. Box-65892, Riyadh, 11566 Saudi Arabia; 2 Department of Mathematics, COMSATS University Islamabad, Lahore Campus, Lahore, Pakistan

**Keywords:** Computational science, Theoretical chemistry

## Abstract

Metal–organic frameworks (MOFs) are vital in modern material science, offering unique properties for gas storage, catalysis, and drug delivery due to their highly porous and customizable structures. Chemical graph theory emerges as a critical tool, providing a mathematical model to represent the molecular structure of these frameworks. Topological indices/molecular descriptors are mathematical formulations applied to molecular models, enabling the analysis of physicochemical properties and circumventing costly lab experiments. These descriptors are crucial for quantitative structure-property and structure-activity relationship studies in mathematical chemistry. In this paper, we study the different molecular descriptors of tetracyanobenzene metal–organic framework. We also give numerical comparison of computed molecular descriptors.

## Introduction

Metal–organic Frameworks, generally called MOFs, constitute a class of compounds that have captivated the research community due to their unique characteristics and applications in various fields^1–3^. As a hybrid of inorganic and organic materials, MOFs are characterized with the aid of their crystalline structures, which are composed of metal ions or clusters coordinated to natural ligands. This combination outcomes in a highly porous fabric with a giant internal surface area, often as compared to the size of a football field within a gram of material. As of now, the Cambridge Structural Database (CSD) has recorded a total of 10,636 synthesized metal–organic Framework (MOF) crystals, along with approximately 114,373 structures that resemble MOFs^4^. Previous initiatives have involved building extensive databases of MOFs and implementing automated, high-throughput processes like molecular simulation and machine learning to forecast the properties of MOFs. These MOF databases comprise actual synthesized and theoretical, or hypothesized, MOF structures^5^.

The defining properties of MOFs is their molecular structures, that’s modular and particularly tunable^6^. At the center, those frameworks encompass metallic nodes, which may be single ions, clusters of ions, or even nanoparticles. These nodes are connected with the aid of natural molecules, regularly referred to as linkers, that are normally composed of carbon, hydrogen, oxygen, and from time-to-time nitrogen^7^. The choice of metal and linker sorts gives upward thrust to a numerous range of MOFs, every with its unique set of properties. These structures aren’t simplest noteworthy for their porosity, however, also for their normal, regularly symmetrical geometries, which can be leveraged in diverse applications.

The synthesis of MOFs entails the self-meeting of steel ions and organic linkers beneath precise conditions. This technique may be stimulated by using factors like temperature, solvent, and pH. Despite the advancements in synthesizing MOFs, challenges remain, in particular in controlling the structure and capability of these frameworks^8^. The crystallization technique main to the formation of MOFs is complicated and now not completely understood, making it tough to predict the outcomes of synthesis with truth^9^.

The MOFs have various uses and applications. One of their most important application is in fuel storage and separation. Due to their excessive porosity, MOFs can keep huge volumes of gases like hydrogen or methane, making them promising materials for clean electricity packages. In carbon capture, MOFs are explored for their capacity to selectively absorb carbon dioxide from commercial emissions, a critical step in fighting climate change^10–12^.

In catalysis, MOFs function as catalysts or catalysts supports. Their well-defined structure offers specific environments for chemical reactions, which can be fantastic in developing more efficient and selective catalysts^13^. MOFs also are being researched for their uses in drug delivery. Their porous nature permits them to encapsulate pharmaceuticals, doubtlessly permitting targeted delivery and controlled release of drugs^14,15^.

Moreover, MOFs has various applications in sensing and detection. Their systems can be designed to change within the presence of precise molecules, making them beneficial in detecting pollution, pollutants, or even biomarkers for sicknesses. In electrochemical power garage, MOFs are being investigated to be used in batteries and supercapacitors. Their structure can contribute to the efficiency and ability of those electricity storage devices^16^.

Chemical structures are every so often considered as complex and vast. They can be challenging to analyze in their natural forms. However, mathematical analysis, particularly graph theory, has been instrumental in simplifying and interpreting these structures in different scientific fields. In graph theory, atoms are represented as vertices and the connections between them as edges, transforming complex chemical or molecular structures into more comprehensible forms.

A topological index is used in chemical graph theory to describe a molecular structure. A topological descriptor reflects important structural characteristics of molecules. Most importantly, it is a structural invariant, meaning its value does not change regardless of how the chemical graph is labeled or depicted. These descriptors have found extensive use in correlating and predicting various chemical properties^17,18^. Topological indices are increasingly used in the development of molecules with pharmacological benefits. See references^19–22^ for further information.

Gutman and Trinajstc introduced the degree-based topological descriptors, known as the first and second Zagreb indices^23^. Since then, various such descriptors have been defined. In 1975, Randić proposed the Randić index $R_{-1/2}$^24^. This concept was later expanded by Bollobás and Erdős^25^, who generalized the Randić index for any real number $\alpha$. Further contributions include the atom bond connectivity index by Estrada et al.^26^, and the geometric arithmetic index *GA*, introduced by Vukicevic et al.^27^. In 2008, Doslic defined the first and second Zagreb coindices^28^. Additionally, Zhou and Trinajsti’c presented the general sum-connectivity index in^29^, refining their earlier sum-connectivity index described in^30^. For recent work, we refer to see^31–34^.

Consider a graph ${\chi }$ with a vertex set $V\left( {\chi }\right)$ comprising ${n}_{\chi }$ vertices and an edge set $E\left( {\chi }\right)$ containing ${m}_{\chi }$ edges. The degree of a vertex ${\lambda _u}$ is the count of edges incident to it. The edges of ${\chi }$ can be categorized based on the degrees of their end vertices, denoted as $\left( {\lambda }_u,{\lambda }_v\right)$, where ${\lambda }_u$ and ${\lambda }_v$ are the degrees of the end vertices *u* and *v*, respectively.

Introduced by Gutman and Trinajstic in 1972^23^, and Das and Gutman^35^ the first and second Zagreb indices respectively of ${\chi }$ are given by:

1 $$\begin{aligned} M_1\left( {\chi }\right) =\sum _{uv \in E\left( {\chi }\right) } (\lambda _u+\lambda _v).\end{aligned}$$

2 $$\begin{aligned} M_2\left( {\chi }\right) =\sum _{uv \in E\left( {\chi }\right) } (\lambda _u\times \lambda _v).\end{aligned}$$

In 2008, Doślić defined the first and second Zagreb coindices^36^:

3 $$\begin{aligned} \overline{M_1}\left( {\chi }\right) =\sum _{uv \notin E\left( {\chi }\right) }[\lambda _u+\lambda _v].\end{aligned}$$

4 $$\begin{aligned} \overline{M_2}\left( {\chi }\right) =\sum _{uv \notin E\left( {\chi }\right) }(\lambda _u\times \lambda _v).\end{aligned}$$

In 2016, Gutman et al. established a relationship to measure Zagreb coindices for a graph ${\chi }$ with $n_{\chi }$ vertices and $m_{\chi }$ edges^37^:

5 $$\begin{aligned} \overline{M_1}\left( {\chi }\right) = 2m_{\chi }\bigg (n_{\chi } -1\bigg ) - M_1\left( {\chi }\right) .\end{aligned}$$

6 $$\begin{aligned} \overline{M_2}\left( {\chi }\right) = 2m_{\chi }^2 -\frac{1}{2} M_1\left( {\chi }\right) -M_2\left( {\chi }\right) .\end{aligned}$$

In 2013, Shirdel et al. introduced the Hyper-Zagreb index^38^:

7 $$\begin{aligned} HM\left( {\chi }\right) =\sum _{uv\in E\left( {\chi }\right) }\big [\lambda _u+\lambda _v\big ]^{2}.\end{aligned}$$

Ghorbani and Azimi in 2012^39^, and Ranjini et al. in 2013^40^, defined the first and second multiple Zagreb indices and the redefined first, second, and third Zagreb indices, respectively:

8 $$\begin{aligned} PM_{1}\left( {\chi }\right) =\prod _{uv\in E\left( {\chi }\right) }[\lambda _u+\lambda _v].\end{aligned}$$

9 $$\begin{aligned} PM_{2}\left( {\chi }\right) =\prod _{uv\in E\left( {\chi }\right) }[\lambda _u\times \lambda _v].\end{aligned}$$

10 $$\begin{aligned} ReZG_{1}\left( {\chi }\right) = \sum _{uv\in E\left( {\chi }\right) }\frac{\lambda _u+\lambda _v}{\lambda _u\times \lambda _v}.\end{aligned}$$

11 $$\begin{aligned} ReZG_{2}\left( {\chi }\right) = \sum _{uv\in E\left( {\chi }\right) }\frac{\lambda _u\times \lambda _v}{\lambda _u+\lambda _v}.\end{aligned}$$

12 $$\begin{aligned} ReZG_{3}\left( {\chi }\right) = \sum _{uv\in E\left( {\chi }\right) } \lambda _u\times \lambda _v(\lambda _u+\lambda _v).\end{aligned}$$

Gutman et al. in 2014 introduced the reduced second Zagreb index^41^:

13 $$\begin{aligned} RM_2\left( {\chi }\right) =\sum _{uv\in E\left( {\chi }\right) }{(\lambda _u-1)(\lambda _v-1)}.\end{aligned}$$

In 2010, Ghorbani and Hosseinzadeh introduced a third version of the Zagreb index^42^:

14 $$\begin{aligned} M_3\left( {\chi }\right) =\sum _{uv\in E\left( {\chi }\right) }(\lambda _u-\lambda _v).\end{aligned}$$

Lastly, the generalized Zagreb index, as defined by Azari and Iranmanesh^43^:

15 $$\begin{aligned} M_{r,s}\left( {\chi }\right) =\sum _{uv\in E\left( {\chi }\right) }(\lambda _u^r\times \lambda _v^s+\lambda _u^s\times \lambda _v^r).\end{aligned}$$

An essential aspect of this study is to address the problem of predicting the physical properties of TCNB MOFs through topological analysis. This analysis is pivotal in understanding the fundamental properties of these frameworks and plays a crucial role in the design and synthesis of similar compounds. By leveraging the calculated topological indices, we aim to create a more efficient pathway for synthesizing MOFs with desired properties. Additionally, this study enriches scientific databases with detailed topological data, facilitating further research in the field. The topological indices calculated in this study are instrumental in predicting various physical properties of TCNB MOFs, such as thermal stability, porosity, and electrical conductivity. These indices serve as a quantitative measure that can guide the synthesis of new MOFs with tailored properties. Furthermore, including these topological data in scientific databases enhances the scope of computational research, offering a valuable resource for researchers exploring the vast potential of MOFs.

### Tetracyanobenzene metal–organic framework (TCNB MOFs)

Tetracyanobenzene metal–organic framework (TCNB MOFs) is a type of porous material, synthesized by reacting a metal salt with TCNB in a solvent, where metal ions coordinate with the cyano groups on TCNB molecules to form an interconnected network. Characterized by high porosity, thermal stability, and electrical conductivity, these MOFs

**Figure 1:**
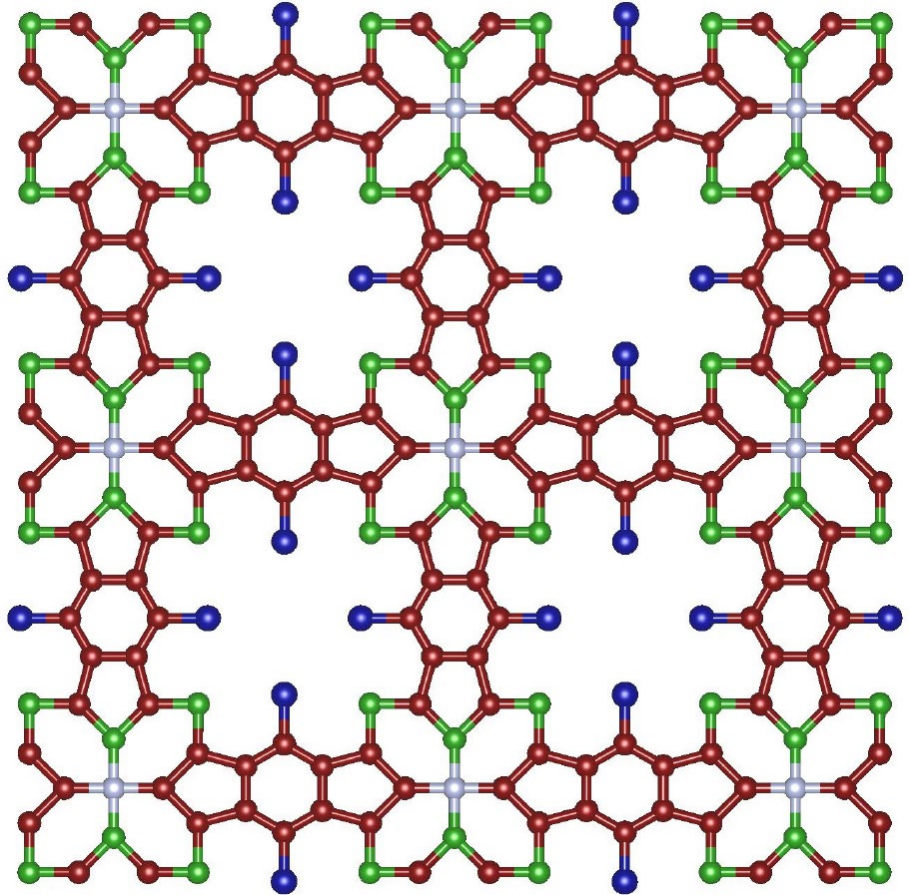
tetracyanobenzene(TCNB).

are versatile in applications like gas storage, catalysis, and energy storage. The *TCNB* framework, a two-dimensional network involving transition metal ions (*TM*) like *Ti*, *V*, *Cr*, *Fe*, *Co*, *Ni*, *Cu*, and *Zn* with tetracyanobenzene, exhibits a unique structure that varies from metallic to half-metallic. Metals such as *Ni*, *Fe*, *Zn*, and *Co* result in fully metallic structures, while *Ti*, *V*, *Cr*, and *Mn* create half-metallic MOFs, showing a gap in one spin direction. The arrangement in the *TCNB* network, especially with *Ti*, *V*, *Cr*, and *Co*, displays a screening effect, where the properties of these metals are significantly influenced by spin-polarized electrons from the surrounding organic ligands, unlike in $TM-Pc$. This effect is absent in $Ni-TCNB$ and $Zn-TCNB$ structures^44–46^.

## Results and discussion

The chemical structure of *TCNB*(*m*, *n*),  depicted in Fig. 1, exhibits a horizontal expansion of ${m}\ge 1$ and a vertical expansion of ${n}\ge 1$. This structure comprises a total of $25{m}+25{n}+32{m}{n}+17$ atoms and $32{m}+32{n}+44{m}{n}+20$ bonds. It features four distinct atom types, categorized by degrees ranging from 1 to 4, with further specifics provided in Table 1. Additionally, the structure includes five different bond types, classified based on the degrees of both end atoms, as elaborated in Table 2.

**Table 1:** Vertex partition of *TCNB*(*m*, *n*).

$\lambda _u$	Frequency	Set of Vertices
1	$2m+2n+4mn$	$V_{1}$
2	$8m+8n+4mn+12$	$V_{2}$
3	$14m+14n+24mn+4$	$V_{3}$
4	$m+n+mn+1$	$V_{4}$

**Table 2:** Edge partition of *TCNB*(*m*, *n*).

$(\lambda _u,\ \lambda _v)$	Frequency	Set of Edges
(1, 3)	$2m+2n+4mn$	$E_{1}$
(2, 3)	$8m+8n+8mn+8$	$E_{2}$
(2, 2)	$4m+4n+8$	$E_{3}$
(3, 3)	$14m+14n+28mn$	$E_{4}$
(3, 4)	$4m+4n+4mn+4$	$E_{5}$

**First Zagreb Index of** *TCNB*(*m*, *n*) We will compute the first Zagreb index of *TCNB*(*m*, *n*),  by using the formula:

$$\begin{aligned}M_1\left( {TCNB(m,n)}\right) =\sum _{uv \in E\left( {TCNB(m,n)}\right) } (\lambda _u+\lambda _v).\end{aligned}$$

Tetracyanobenzene ($TCNB\left( m,n\right)$) structure consist of five types of edge partitions, given in Table 2. By using these values, we have

$$\begin{aligned} M_{1}\left( TCNB\left( m,n\right) \right)&=(2m+2n+4mn)(1+3)+(8m+8n+8mn+8)(2+3)\\ & \quad +(4m+4n+8)(2+2)+(14m+14n+28mn)(3+3)\\ &\quad +(4m+4n+4mn+4)(3+4)\\&=176m+176n+252mn+100. \end{aligned}$$

**Second Zagreb Index of** *TCNB*(*m*, *n*) Second Zagreb index of $TCNB\left( m,n\right)$ is

$$\begin{aligned} M_{2}\left( TCNB\left( m,n\right) \right)&=(2m+2n+4mn)(1\times 3)+(8m+8n+8mn+8)(2\times 3)\\ &\quad +(4m+4n+8)(2\times 2)+(14m+14n+28mn)(3\times 3)\\ &\quad +(4m+4n+4mn+4)(3\times 4)\\&=244m+244n+360mn+38059. \end{aligned}$$

**First and Second Zagreb Coindices of** *TCNB*(*m*, *n*) First Zagreb coindex of *TCNB*(*m*, *n*) is

$$\begin{aligned} {\overline{M}}_{1}\left( TCNB\left( m,n\right) \right)&=2(32m+32n+44mn+20)(25m+25n+33mn+17-1)\\&\quad -(176m+176n+252mn+100)\\&=2904m^2n^2+4312m^2n+1600m^2+4312mn^2+5676mn+1848m\\ &\quad +1600n^2+1848n+540. \end{aligned}$$

Second Zagreb Coindex of *TCNB*(*m*, *n*) is

$$\begin{aligned} {\overline{M}}_{2}\left( TCNB\left( m,n\right) \right)&=2(32m+32n+44mn+20)^2-\left( \frac{1}{2}\right) (176m+176n+252mn+100)\\ &\quad -(244m+244n+360mn+380)\\ &=3872m^2n^2+5632m^2n+2048m^2+5632mn^2+3170mn+2228m\\ &\quad +2048n^2+2228n+370. \end{aligned}$$

**Hyper Zagreb Index of** *TCNB*(*m*, *n*) Now, we will compute the hyper Zagreb index of *TCNB*(*m*, *n*) by using the equation below and partition given in Table 2.

$$\begin{aligned}HM\left( {TCNB(m,n)}\right)= & \sum _{uv\in E\left( {TCNB(m,n)}\right) }\big [\lambda _u+\lambda _v\big ]^{2}.\\ HM\left( TCNB\left( m,n\right) \right)= & (2m+2n+4mn)(1+3)^2+(8m+8n+8mn+8)(2+3)^2\\ & \quad +(4m+4n+8)(2+2)^2+(14m+14n+28mn)(3+3)^2\\ & \quad +(4m+4n+4mn+4)(3+4)^2,\\ = & 996m+996n+1468mn+524. \end{aligned}$$

**First and Second Multiplicative Zagreb Indices of** *TCNB*(*m*, *n*) Edge types for tetracyanobenzene transition metal organic network are defined in Table 2. Again using these values, we calculated the first multiplicative Zagreb index of *TCNB*(*m*, *n*) is:

$$\begin{aligned}&PM_{1}\left( {TCNB(m,n)}\right) =\\& \prod _{uv\in E\left( {TCNB(m,n)}\right) }[\lambda _u+\lambda _v].\\& =(1+3)^{2m+2n+4mn}\times (2+3)^{8m+8n+8mn+8}\\&\quad \times (2+2)^{4m+4n+8}\times (3+3)^{14m+14n+28mn}\\&\quad \times (3+4)^{4m+4n+4mn+4},\\& =4^{6m+6n+4mn+8}\times 5^{8m+8n+8mn+8}\times 6^{14m+14n+28mn}\times 7^{4m+4n+4mn+4}. \end{aligned}$$

Second multiplicative Zagreb indices of *TCNB*(*m*, *n*) is:

$$\begin{aligned}&PM_{2}\left( TCNB\left( m,n\right) \right) =\\& \prod _{uv\in E\left( {TCNB(m,n)}\right) }[\lambda _u\times \lambda _v].\\& =(1\times 3)^{2m+2n+4mn}\times (2\times 3)^{8m+8n+8mn+8}\\&\quad \times (2\times 2)^{4m+4n+8}\times (3\times 3)^{14m+14n+28mn}\\&\quad \times (3\times 4)^{4m+4n+4mn+4},\\& =3^{42m+42n+26mn+68}\times 2^{24m+24n+26mn+32}. \end{aligned}$$

**The first, second, and third redefined Zagreb indices of** *TCNB*(*m*, *n*)

$$\begin{aligned}&ReZG_{1}\left( TCNB\left( m,n\right) \right) =\\& \sum _{uv\in E\left( {TCNB(m,n)}\right) }\frac{\lambda _u+\lambda _v}{\lambda _u\times \lambda _v}=\\& =(2m+2n+4mn)\left( \frac{1+3}{1\times 3}\right) +(8m+8n+8mn+8)\left( \frac{2+3}{2\times 3}\right) \\& \quad +(4m+4n+8)\left( \frac{2+2}{2\times 2}\right) +(14m+14n+28mn)\left( \frac{3+3}{3\times 3}\right) \\&\quad +(4m+4n+4mn+4)\left( \frac{3+4}{3\times 4}\right) \\ & =25m+25n+33mn+17. \\&\quad ReZG_{2}\left( TCNB\left( m,n\right) \right) =\\& \sum _{uv\in E\left( {TCNB(m,n)}\right) }\frac{\lambda _u\times \lambda _v}{\lambda _u+\lambda _v}=\\& \left( 2m+2n+4mn\right) \left( \frac{1\times 3}{1+3}\right) +\left( 8m+8n+8mn+8\right) \left( \frac{2\times 3}{2+3}\right) \\&\quad +\left( 4m+4n+8\right) \left( \frac{2\times 2}{2+2}\right) +\left( 14m+14n+28mn\right) \left( \frac{3\times 3}{3+3}\right) \\&\quad +\left( 4m+4n+4mn+4\right) \left( \frac{3\times 4}{3+4}\right) \\ & =42.9571m+42.9571n+61.4571mn+24.4571. \\&\quad ReZG_{3}\left( TCNB\left( m,n\right) \right) =\\& \sum _{uv\in E\left( {TCNB(m,n)}\right) } \lambda _u\times \lambda _v(\lambda _u+\lambda _v)=\\& (2m+2n+4mn)(1\times 3)(1+3)+(8m+8n+8mn+8)(2\times 3)(2+3)\\&\quad +(4m+4n+8)(2\times 2)(2+2)+(14m+14n+28mn)(3\times 3)(3+3)\\&\quad (4m+4n+4mn+4)(3\times 4)(3+4)\\ &=1420m+1420n+2136mn+704. \end{aligned}$$

**The Reduced Second Zagreb index of** *TCNB*(*m*, *n*)

$$\begin{aligned}&RM_{2}\left( TCNB\left( m,n\right) \right) =\\& =\sum _{uv\in E\left( {TCNB(m,n)}\right) }{(\lambda _u-1)(\lambda _v-1)}\\& =\left( 2m+2n+4mn\right) (1-1)(3-1)+\left( 8m+8n+8mn+8\right) (2-1)(3-1)\\&\quad +\left( 4m+4n+8\right) (2-1)(2-1)+\left( 14m+14n+28mn\right) (3-1)(3-1)\\&\quad +\left( 4m+4n+4mn+4\right) (3-1)(4-1)\\ &=100m+100n+152mn+48. \end{aligned}$$

**The Third Zagreb index of** *TCNB*(*m*, *n*)

$$\begin{aligned}&M_{3}\left( TCNB\left( m,n\right) \right) \\& =\sum _{uv\in E\left( {TCNB(m,n)}\right) }(\lambda _u-\lambda _v)\\& =\left( 2m+2n+4mn\right) (1-3)+\left( 8m+8n+8mn+8\right) (2-3)\\&\quad +\left( 4m+4n+8\right) (2-2)+\left( 14m+14n+28mn\right) (3-3)\\&\quad +\left( 4m+4n+4mn+4\right) (3-4)\\ &=-16m-16n-20mn-12. \end{aligned}$$

**The Generalized Zagreb index of** *TCNB*(*m*, *n*)

$$\begin{aligned}&M_{r,s}\left( TCNB\left( m,n\right) \right) \\& =\sum _{uv\in E\left( {TCNB(m,n)}\right) }(\lambda _u^r\times \lambda _v^s+\lambda _u^r\times \lambda _v^s)\\& =(4m+4n+8mn)(1^r\times 3^s)+(16m+16n+16mn+16)(2^r\times 3^s)\\&\quad +(8m+8n+16)(2^{r}+2^{s})+(28m+28n+56mn)(3^{r}+3^{s})\\&\quad +(8m+8n+8mn+8)(3^r\times 4^s)\\ & =(4m+4n+8mn)(1^r\times 3^s)\\&\quad +(16m+16n+16mn+16)(2^r\times 3^s)\\&\quad +(8m+8n+16)(2^{r+s})+(28m+28n+56mn)(3^{r+s})\\&\quad +(8m+8n+8mn+8)(3^r\times 4^s). \end{aligned}$$

### Ethical approval

This article does not contain any studies with human participants or animals performed by any of the authors.

## Comparative discussion and conclusion

In this study, we comprehensively analyzed various degree and neighborhood-based topological indices of the tetracyanobenzene transition metal organic network, $TCNB (m, n)$. This investigation, focusing on analytical results, highlighted the intricate relationships between these indices and the structural properties of the TCNB network, thereby revealing their potential applications in diverse scientific and industrial fields.

The correlations observed between the topological indices and the physical properties of TCNB MOFs, such as thermal stability, porosity, and electrical conductivity, underscore the utility of topological analysis in predicting MOF behavior and properties. This is pivotal for synthesizing new materials with desired characteristics and contributes significantly to the field of material science, particularly in designing MOFs for specific applications like gas storage, catalysis, or drug delivery. Employing mathematical methods, especially those from chemical graph theory, has allowed for a more efficient and accurate prediction of MOFs’ physical properties. By simplifying complex molecular structures into understandable forms, these methods have proven effective in providing a quantitative framework for interpreting MOFs’ structural attributes. The application of topological indices in MOF analysis presents promising research, opening possibilities for developing more efficient methods for predicting material properties and designing new materials with customized functionalities. Future research could extend this approach to other types of MOFs, further enhancing the predictive models for material properties and contributing to the advancement of computational research in this domain.

**Figure 2:**
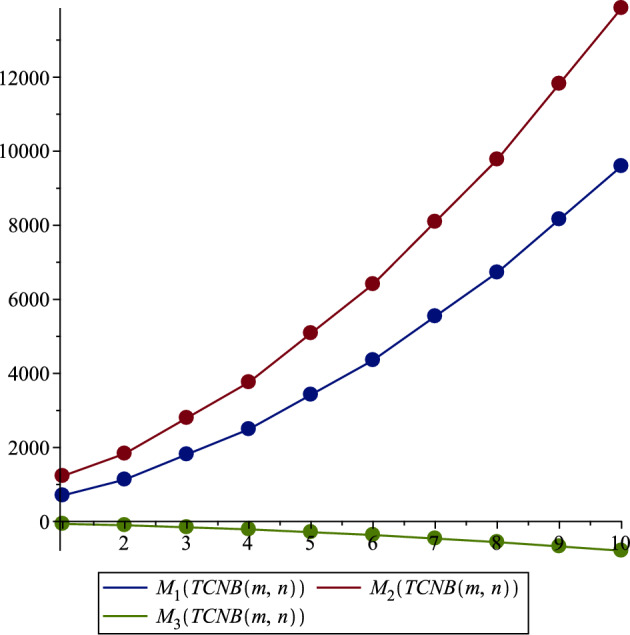
Zagreb indices.

**Figure 3:**
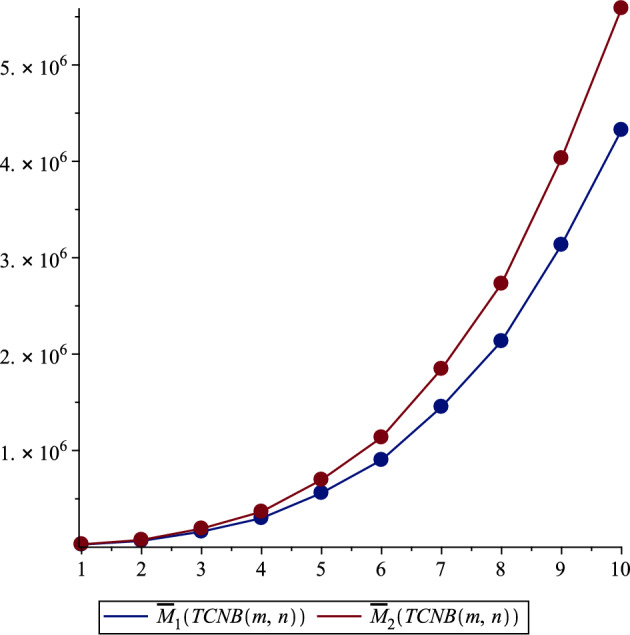
Zagreb co-indices.

**Figure 4:**
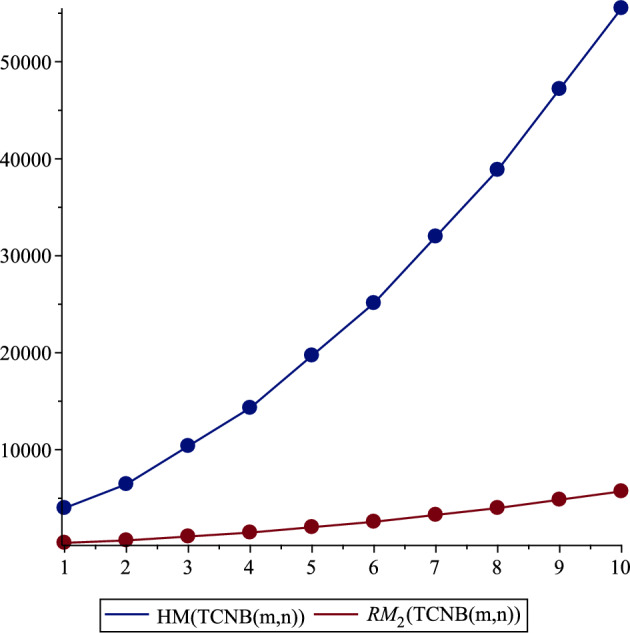
Hyper and reduced second Zagreb indices.

**Figure 5:**
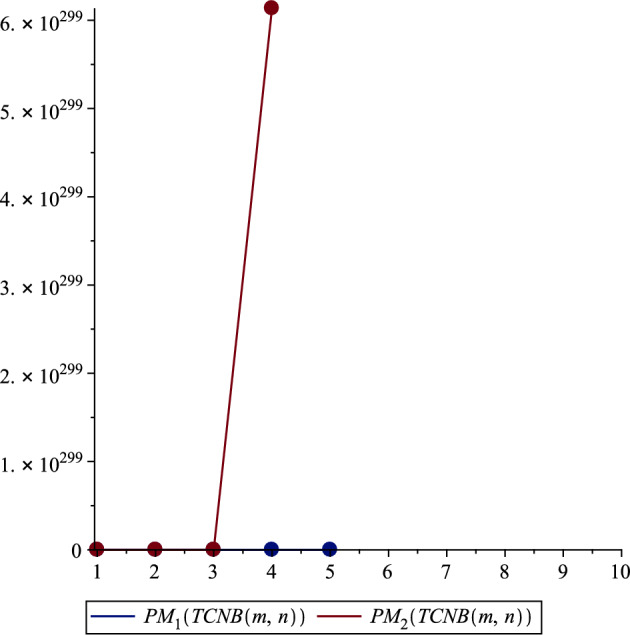
Multiple Zagreb indices.

**Figure 6:**
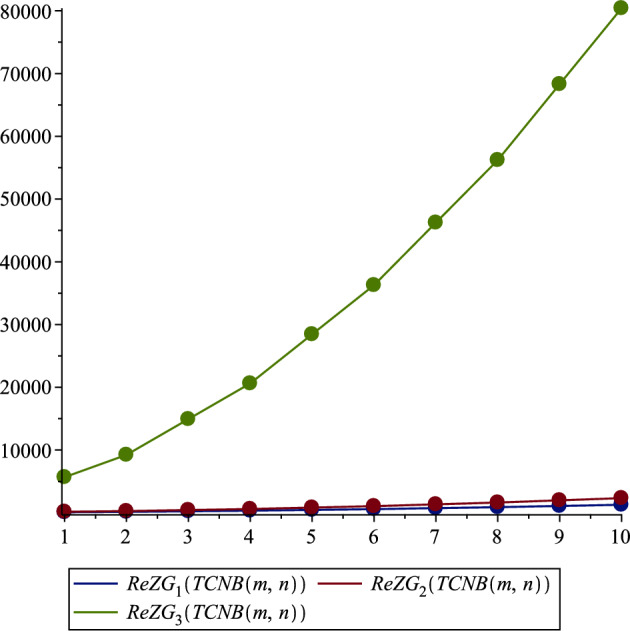
Reduced Zagreb indices.

**Figure 7:**
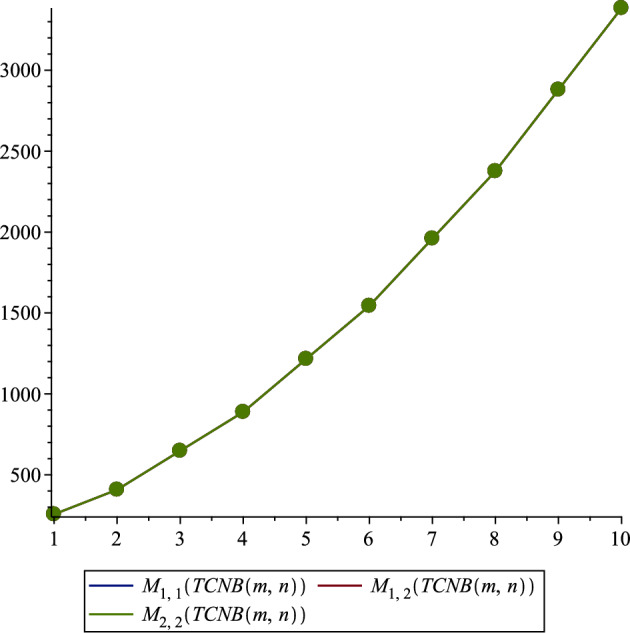
Generalized Zagreb indices for different values of *r*, *s*.

In our comprehensive analysis of the Tetracyanobenzene metal–organic Frameworks (TCNB MOFs), symbolized as $TCNB (m, n)$, we observed consistent trends across various topological indices, as detailed in Tables 3, 4, 5, 6, 7, and 8, and shown in Figs. 2, 3, 4, 5, 6 and 7. For the first Zagreb index ($M_1$), values began at 704.0 for $TCNB (1,1)$ and increased progressively, reaching 9596.0 for $TCNB (5,6)$ as shown in Table 3. This pattern was mirrored in the second Zagreb index ($M_2$), which started at 1228.0 for $TCNB (1,1)$ and escalated to 13864.0 for $TCNB (5,6)$, also documented in Table 3. Interestingly, the first Zagreb index values were consistently lower than the second Zagreb index but higher compared to the third Zagreb index ($M_3$), which started at -64.0 for $TCNB (1,1)$ and decreased further to -788.0 for $TCNB (5,6)$, as depicted in Table 3.

Similarly, for the Zagreb coindices (${\overline{M}}_1$ and ${\overline{M}}_2$), there was a notable increase in values as the size of $m, n$ increased, as can be seen in Table 4. For instance, ${\overline{M}}_1$ for $TCNB (1,1)$ was 24640.0, escalating significantly to 4325300.0 for $TCNB (5,6)$. This trend of increasing values was consistent across all topological indices studied, including the Hyper Zagreb Index ($HM$) and Reduced Zagreb Indices ($ReZG_1, ReZG_2, ReZG_3$) as presented in Tables 5 and 7, as well as the Multiple Zagreb Indices ($PM_1, PM_2$) and Generalized Zagreb Indices ($M_{1,1}, M_{1,2}, M_{2,2}$) shown in Tables 6 and 8. Each of these indices showed a clear pattern of escalation with the increase in the size of the MOF network, reflecting the growing complexity and interaction within the TCNB MOFs.

**Table 3:** Comparison of Zagreb first, second and third indices.

${\varvec{\left( m,n\right) }}$	${\varvec{M_1\left( TCNB\left( m,n\right) \right) }}$	${\varvec{M_2\left( TCNB\left( m,n\right) \right) }}$	${\varvec{M_3\left( TCNB\left( m,n\right) \right) }}$
$\left( 1,1\right)$	704.0	1228.0	$-64.0$
$\left( 1,2\right)$	1132.0	1832.0	$- 100.0$
$\left( 2,2\right)$	1812.0	2796.0	$- 156.0$
$\left( 2,3\right)$	2492.0	3760.0	$- 212.0$
$\left( 3,3\right)$	3424.0	5084.0	$- 288.0$
$\left( 3,4\right)$	4356.0	6408.0	$- 364.0$
$\left( 4,4\right)$	5540.0	8092.0	$- 460.0$
$\left( 4,5\right)$	6724.0	9776.0	$- 556.0$
$\left( 5,5\right)$	8160.0	11820.0	$- 672.0$
$\left( 5,6\right)$	9596.0	13864.0	$- 788.0$

**Table 4:** Comparison of Zagreb first and second co-indices.

${\varvec{ (m,n )}}$	${\varvec{{\overline{M}}_1 (TCNB (m,n ) )}}$	${\varvec{{\overline{M}}_2 (TCNB (m,n ) )}}$
$\left( 1,1\right)$	24640.0	27228.0
$\left( 1,2\right)$	62924.0	72914.0
$\left( 2,2\right)$	158890.0	190410.0
$\left( 2,3\right)$	298540.0	365510.0
$\left( 3,3\right)$	559580.0	696890.0
$\left( 3,4\right)$	901970.0	1135900.0
$\left( 4,4\right)$	1452700.0	1846600.0
$\left( 4,5\right)$	2134100.0	2730400.0
$\left( 5,5\right)$	3133900.0	4032300.0
$\left( 5,6\right)$	4325300.0	5588300.0

**Table 5:** Comparison of Hyper Zagreb and reduced second Zagreb indices.

${\varvec{(m,n)}}$	${\varvec{HM(}}{{\varvec{TCNB(m,n))}}}$	${\varvec{RM}}_{\varvec{2}}\left( {\varvec{TCNB}}\left( {\varvec{m,n}}\right) \right)$
$\left( 1,1\right)$	3984.0	400.0
$\left( 1,2\right)$	6448.0	652.0
$\left( 2,2\right)$	10380.0	1056.0
$\left( 2,3\right)$	14312.0	1460.0
$\left( 3,3\right)$	19712.0	2016.0
$\left( 3,4\right)$	25112.0	2572.0
$\left( 4,4\right)$	31980.0	3280.0
$\left( 4,5\right)$	38848.0	3988.0
$\left( 5,5\right)$	47184.0	4848.0
$\left( 5,6\right)$	55520.0	5708.0

**Table 6:** Comparison of Multiple Zagreb first and second indices.

${\varvec{(m,n)}}$	${\varvec{PM}}_{\varvec{1}}{\varvec{(TCNB(m,n))}}$	${\varvec{PM}}_{\varvec{2}}{\varvec{(TCNB(m,n))}}$
$\left( 1,1\right)$	${3.7711\times 10^{43}}$	${ 6.8669\times 10^{116}}$
$\left( 1,2\right)$	${1.8148\times 10^{76}}$	${ 2.1503\times 10^{164}}$
$\left( 2,2\right)$	${5.3630\times 10^{130}}$	${ 1.1486\times 10^{232}}$
$\left( 2,3\right)$	${1.5849\times 10^{185}}$	${ 6.1357\times 10^{299}}$
$\left( 3,3\right)$	${2.8762\times 10^{261}}$	${ 5.5908\times 10^{387}}$
$\left( 3,4\right)$	${5.2197\times 10^{337}}$	${ 5.0943\times 10^{475}}$
$\left( 4,4\right)$	${5.8170\times 10^{435}}$	${ 7.9182\times 10^{583}}$
$\left( 4,5\right)$	${6.4827\times 10^{533}}$	${ 1.2307\times 10^{692}}$
$\left( 5,5\right)$	${4.4366\times 10^{653}}$	${ 3.2632\times 10^{820}}$
$\left( 5,6\right)$	${3.0363\times 10^{773}}$	${ 8.6520\times 10^{948}}$

**Table 7:** Comparison of Reduced Zagreb first, second and third indices.

${\varvec{(m,n )}}$	${\varvec{ReZG}}_{\varvec{1}} {\varvec{(TCNB (m,n ) )}}$	${\varvec{ReZG}}_{\varvec{2 }}{\varvec{(TCNB (m,n ) )}}$	${\varvec{ReZG}}_{\varvec{3}} {\varvec{(TCNB (m,n ) )}}$
$\left( 1,1\right)$	100.0	171.83	5680.0
$\left( 1,2\right)$	158.0	276.24	9236.0
$\left( 2,2\right)$	249.0	442.11	14928.0
$\left( 2,3\right)$	340.0	607.99	20620.0
$\left( 3,3\right)$	464.0	835.31	28448.0
$\left( 3,4\right)$	588.0	1062.6	36276.0
$\left( 4,4\right)$	745.0	1351.4	46240.0
$\left( 4,5\right)$	902.0	1640.2	56204.0
$\left( 5,5\right)$	1092.0	1990.5	68304.0
$\left( 5,6\right)$	1282.0	2340.7	80404.0

**Table 8:** Comparison of Generalized Zagreb indices for different $\left\{ r,s\right\}$.

(*m*, *n*)	${\varvec{M}}_{{\varvec{1,1}}} {\varvec{(TCNB (m,n ) )}}$	${\varvec{M}}_{{\varvec{1,2}}} {\varvec{(TCNB (m,n ) )}}$	${\varvec{M}}_{{\varvec{2,2}}} {\varvec{(TCNB (m,n ) )}}$
$\left( 1,1\right)$	256.0	256.0	256.0
$\left( 1,2\right)$	408.0	408.0	408.0
$\left( 2,2\right)$	648.0	648.0	648.0
$\left( 2,3\right)$	888.0	888.0	888.0
$\left( 3,3\right)$	1216.0	1216.0	1216.0
$\left( 3,4\right)$	1544.0	1544.0	1544.0
$\left( 4,4\right)$	1960.0	1960.0	1960.0
$\left( 4,5\right)$	2376.0	2376.0	2376.0
$\left( 5,5\right)$	2880.0	2880.0	2880.0
$\left( 5,6\right)$	3384.0	3384.0	3384.0

## Data Availability

The data that support the findings of this study are available in the manuscript.
